# Beyond the Baroreflex: A New Measure of Autonomic Regulation Based on the Time-Frequency Assessment of Variability, Phase Coherence and Couplings

**DOI:** 10.3389/fnetp.2022.891604

**Published:** 2022-06-06

**Authors:** Philip T. Clemson, Jeffrey B. Hoag, William H. Cooke, Dwain L. Eckberg, Aneta Stefanovska

**Affiliations:** ^1^ Department of Electrical Engineering and Electronics, University of Liverpool, Liverpool, United Kingdom; ^2^ Physics Department, Lancaster University, Lancaster, United Kingdom; ^3^ Jane and Leonard Korman Respiratory Institute, Thomas Jefferson University, Philadelphia, PA, United States; ^4^ Kinesiology and Integrative Physiology Department, Michigan Technological University, Houghton, MI, United States; ^5^ Departments of Medicine and Physiology, Virginia Commonwealth University School of Medicine, Richmond, VA, United States; ^6^ Department of Veterans Affairs Medical Center, Richmond, VA, United States

**Keywords:** atropine, propranolol, baroreflex, wavelet phase coherence, phase shift

## Abstract

For decades the role of autonomic regulation and the baroreflex in the generation of the respiratory sinus arrhythmia (RSA) - modulation of heart rate by the frequency of breathing - has been under dispute. We hypothesized that by using autonomic blockers we can reveal which oscillations and their interactions are suppressed, elucidating their involvement in RSA as well as in cardiovascular regulation more generally. R-R intervals, end tidal CO_2_, finger arterial pressure, and muscle sympathetic nerve activity (MSNA) were measured simultaneously in 7 subjects during saline, atropine and propranolol infusion. The measurements were repeated during spontaneous and fixed-frequency breathing, and apnea. The power spectra, phase coherence and couplings were calculated to characterise the variability and interactions within the cardiovascular system. Atropine reduced R-R interval variability (*p* < 0.05) in all three breathing conditions, reduced MSNA power during apnea and removed much of the significant coherence and couplings. Propranolol had smaller effect on the power of oscillations and did not change the number of significant interactions. Most notably, atropine reduced R-R interval power in the 0.145–0.6 Hz interval during apnea, which supports the hypothesis that the RSA is modulated by a mechanism other than the baroreflex. Atropine also reduced or made negative the phase shift between the systolic and diastolic pressure, indicating the cessation of baroreflex-dependent blood pressure variability. This result suggests that coherent respiratory oscillations in the blood pressure can be used for the non-invasive assessment of autonomic regulation.

## 1 Introduction

The response of the autonomic nervous system to blood pressure changes or *baroreflex* is central to the function of the cardiovascular system. It is known to modulate the heart rate by altering the activity of the sympathetic branch of the autonomic nervous system (Eckberg et al., 1988). Respiratory sinus arrhythmia has long been proposed as manifesting via the baroreflex (Piepoli et al., 1997; Karemaker, 2009). However, the central cardiac and respiratory generators located in the brainstem have been suggested as an alternate origin of the respiratory sinus arrhythmia (Hirsch and Bishop, 1981; Koepchen et al., 1981; Berntson et al., 1993). Unfortuntately, the interconnectedness and nonlinearity of the systems involved makes distinguishing between these two hypotheses a difficult task (Billman, 2013).

There are two fundamental ways in which one can measure the autonomic control of the cardiovascular system. Firstly, one can measure the baseline offset in the values of the heart rate, vascular tone and blood pressure which are set by the autonomic nerve activity. However, it is difficult to measure such average values reliably given the time-varying nature of the cardiovascular system (Stefanovska et al., 2001; Stefanovska, 2007; Penzel et al., 2017). Alternatively, one can measure the changes in the *variability* of these values. For example, the regulation of the cardiac output by the baroreflex results in Mayer waves of frequency ∼0.1 Hz in the heart rate and blood pressure variability (Malpas, 2002; Julien, 2006). In addition, studies have suggested that the sympathetic influence on the microvascular blood flow is manifested in the 0.021–0.052 Hz interval (Bajrović et al., 2000; Söderström et al., 2003). Other types of dynamics such as temporal asymmetries in the R-R interval variability have also been linked to autonomic regulation (Porta et al., 2008).

By far one of the most common ways of assessing the strength of the autonomic control is the baroreflex sensitvity (Robbe et al., 1987; Rovere et al., 2013). However, studies have revealed problems with the reliability and reproducibiltiy of these measurements (Carrasco-Sosa et al., 2005; Dietricha et al., 2010). In particular, the different mechanisms by which the respiration produces variability in the heart rate and the blood pressure interfere with the measurement of the baroreflex sensitivity (Badra et al., 2001; Carrasco-Sosa et al., 2005; Tiinanen et al., 2008). It has also been shown that the calculation of baroreflex sensitivity using cross correlation does not quantify the causal relationship between the blood pressure variability and heart rate variability (Wessel et al., 2020). Alternative ways of characterising the autonomic control of cardiovascular function are therefore sought.

Other analyses of the autonomic regulation of the cardiovascular system have advanced significantly in the last two decades. The low-frequency (LF)/high-frequency (HF) quotient of R-R interval variability was once widely used to determine the sympathovagal balance (Malliani et al., 1991). However, while the LF (0.15–0.4 Hz) variability was previously identified as a marker of sympathetic activity, this has since been shown to be inaccurate (Randall et al., 1991; Hopf et al., 1995; Eckberg, 1997; Sunagawa et al., 1998; Billman, 2013; Hoshi et al., 2019; Pernice et al., 2019). These studies have instead shown that the cardiovascular system exhibits significant nonlinear behaviour; the effects of the sympathetic activity are modulated by the parasympathetic activity and vice versa. As such, the notion that the autonomic-derived variability can be divided into distinct sympathetic and parasympathetic frequency bands has been rescinded. Furthermore, other oscillations aside from those generated by the autonomic nervous system may also be attributed to the power in these frequency intervals (Penzel et al., 2017). Specifically, the vascular smooth muscle in the walls of the microcirculation exhibits spontaneous oscillations around 0.1 Hz *in vivo* (Salerud et al., 1983; Colantuoni et al., 1984; Intaglietta, 1989), and similar oscillations have been reported in large isolated arteries *in vitro* (Nilsson and Aalkjaer, 2003). Such oscillations have an impact on the vascular resistance and could therefore appear in the R-R interval variability *via* the baroreflex.

Many of these advancements in understanding have come as a result of the development of new mathematical and physical models of the cardiovascular system, along with corresponding time series analysis methods. Previous frameworks based on linear transfer functions revealed the main effects of the autonomic regulation (Saul et al., 1989). However, one of the most important shifts has been the move to nonlinear models and methods to examine more subtle regulatory mechanisms. For example, when investigating the response of the cardiovascular dynamics to sympathetic and parasympathetic blocking drugs, studies have revealed changes in the cardiovascular interactions by performing analyses based on Granger causality (Porta et al., 2013). Spectral analysis methods have also been used to analyse changes in the amplitude and phase of the cardiovascular oscillations (Elstad et al., 2011). However, the wealth of information that can be extracted in the time-frequency domain has been left mostly untouched apart from a handful of studies (Stankovski et al., 2013; de Boer and Karemaker, 2019).

In this work we use a novel approach based on the assumption that the cardiovascular system is nonlinear and mainly deterministic (Stefanovska et al., 2001). This contrasts with the transfer function framework but also the Wiener-Granger causality methods, which describe the system behaviour by using a stochastic process (Porta and Faes, 2016; Faes et al., 2017). In the case of the latter, stochasticity is needed to account for the explicit time-varying nature of the system. In the new approach we instead consider a system of coupled nonlinear, nonautonomous and self-sustained oscillators with time-varying frequencies, phases and couplings. This system models the complex dynamics without the need to assign any of the variability to stochastic effects, leading to a more complete description of the various mechanisms involved (Clemson and Stefanovska, 2014; Clemson et al., 2016). For these time-varying oscillators it is also important to consider their finite-time dynamics (Newman et al., 2021). As such, we also apply time-localised analysis using time-frequency domain methods in order to characterise the dynamics.

In applying this new approach we use time series analysis to decompose the cardiovascular and neuronal dynamics into their oscillatory components. Specifically, we combine time-frequency analysis and information theory methods together with surrogate data techniques. Based on previous work, we apply wavelet-based approaches with logarithmic frequency resolution (Bandrivskyy et al., 2004; Iatsenko et al., 2015; Clemson et al., 2016). To analyse more subtle effects of the nonlinear couplings between different systems we apply a method based on Granger causality to estimate the information transfer between the measured time series (Paluš and Stefanovska, 2003; Vejmelka and Paluš, 2008). As such, we are able to observe changes in the amplitude and phase relations of oscillations but do not miss information about the couplings which are not always obvious in the time-frequency domain (Clemson et al., 2016). Similarly, when analysing the couplings between different systems, phenomena such as phase-locking which are not detectable using the information theoretic approach can still be identified.

We hypothesised that by applying a pharmacological blockade of the autonomic nervous system, the effects of underlying non-autonomic regulatory mechanisms would be revealed in the nonlinear couplings and phase coherence observed between oscillations in the heart rate, respiration, blood pressure and sympathetic nerve activity. In this way, new measures of the autonomic regulation can be established to provide illumination on important interactions such as respiratory sinus arrhythmia.

## 2 Materials and Methods

### 2.1 Subjects

Seven healthy volunteers (six men), with average age ±S.E.M. 29.4 ± 2.3 years; height 173.4 ± 2.4 cm; weight 79.4 ± 3.5 kg were studied after they had abstained from caffeine and exercise for 24 h. All subjects were non-smokers, had no evidence of heart disease, and took no medications. This study was approved by the human research committees of the Hunter Holmes McGuire Department of Veterans Affairs Medical Center and the Medical College of Virginia at Virginia Commonwealth University. All subjects gave their written informed consent prior to participating.

### 2.2 Measurements

Data were recorded simultaneously at 500 Hz with commercial hardware and software (WINDAQ, Dataq Instruments, Akron, OH, United States). An electrocardiogram (ECG), respiration (uncalibrated pnuemobelt), and finger photoplethysmographic arterial pressure (Finapres, Model 2300, Ohmeda, Englewood, CO, United States) were continuously measured. End tidal carbon dioxide (CO_2_) concentrations were measured on a breath-by-breath basis at the mouth (Infrared Analyzer, Gambo Engström, Sweden).

Muscle sympathetic nerve activity was recorded directly (Nerve Traffic Analyzer, Model 662C-1, University of Iowa Bioengineering, Iowa City, IA, United States), as described previously (Wallin and Eckberg, 1982). Briefly, multifiber sympathetic efferent traffic from peroneal nerve muscle fascicles was lead off with tungsten microelectrodes with uninsulated tip diameters of about 2 μm. A reference electrode was inserted subcutaneously 1–2 cm from the recording electrode. Both electrodes were connected to a differential preamplifier, and then to an amplifier (total gain of 70,000) where the nerve signal was band-pass filtered (700–2000 Hz), and integrated (time constant 0.1 s) to obtain mean voltage neurograms. Satisfactory recordings of sympathetic nerve activity were defined by pulse synchronous bursts that increased during end-expiratory apnea or Valsalva straining, and did not change during tactile or auditory stimulation.

### 2.3 Experimental Protocol

The subjects were asked to follow three protocols: 1) subjects rested quietly, and breathed spontaneously (uncontrolled frequency) for five minutes; 2) subjects controlled their breathing rate at fifteen breaths per minute (metronome) for five minutes; and 3) subjects voluntarily hyperventilated while breathing 100% oxygen for 2 min, inspired, and held their breath for as long as possible. The protocol was repeated following a saline control, then a complete parasympathetic (atropine sulfate, 0.04 mg/kg, intravenous) blockade, followed by a sympathetic (propranolol, 0.2 mg/kg, intravenous) blockade (hereafter referred to as the *double* blockade). On a separate day, each subject returned for the saline control and sympathetic blockade only. Sympathetic nerve activity was analyzed with custom programs developed for use with commercial software (WINDAQ, Dataq Instruments, Akron, OH, United States). Bursts with a signal-to-noise ratio > 3:1, and latencies from preceding R waves of about 1.3 s were automatically detected (Fagius et al., 1987). One observer manually over-read results of automated analyses. The measurements were continuous between the stages of the experimental protocol.

### 2.4 Data Analysis

#### 2.4.1 Preprocessing

The low-frequency content of the ECG signals was suppressed by subtracting a 0.1 s moving average. The R-peaks were then identified as peaks which exceeded a threshold of two standard deviations above the mean of the samples. Ectopic beats and noise artifacts in the extracted R-peak series were detected from the estimate of the derivative of the instantaneous heart rate using the method proposed by Mateo and Laguna (2003). When an ectopic/noise—generated beat was found, the two affected R-R intervals on either side of the beat were linearly interpolated over. The median percentage of ectopic beats across the subjects was 0.101% with a range of 0.077–0.136%. Continuous systolic and diastolic pressures were delineated from the onsets, systolic peaks and dicrotic notches for each pulse wave in the pressure signal (Li et al., 2010). The CO_2_ signal was used to analyse the respiration dynamics. The raw sympathetic nerve activity signals were used to reduce the dependence of the analysis on peak and burst-detection algorithms with arbitrary parameter selections. Between-subject amplitude effects in the nerve activity signals were nullified by the baseline measurements using saline and the within-subject statistics performed in the analysis. The analytical methods were applied to the entirety of the recordings to reduce the influence of edge effects. Lastly, the lowest-frequency components from each signal were removed over the entire recording session by subtracting a 50 s moving average. This detrending was performed so that unobservable low-frequency components of the signals did not influence the results based on the higher-frequency dynamics, e.g. due to second and third harmonics of the low-frequency components, or the effective baseline offset they produce in shorter time windows.

#### 2.4.2 Optimised Time-Frequency Representation of the Signals—The Wavelet Transform

The continuous Morlet wavelet transform was applied to each signal to track the change in the dynamics for each part of the protocol, using the method described in an earlier paper (Stefanovska et al., 1999). The wavelet transform provides a 3-dimensional space to detect the power and frequency of oscillations at all times in a signal. Its logarithmic frequency resolution also allows lower-frequency oscillations to be distinguished, which might otherwise be included in a single wider frequency band.

The power of the wavelet transform *W*_*T*_(*f*_*k*_, *t*) for a given frequency *f*_*k*_, within a time interval denoted by *j* was found using

$$P_{j}\left(f_{k}\right)=\left(f_{k}-f_{k-1}\right){\left(\frac{1}{n_{j}^{\prime }-n_{j}+1}\overset{n_{j}^{\prime }}{\underset{n=n_{j}}{\sum }}|W_{T}\left(f_{k},t_{n}\right)|\right)}^{2},$$

where *f*_*k*_ is an ascending logarithmic frequency scale, *t*_*n*_ is the sample time and *n*_*j*_ and $n_{j}^{\prime }$ are the indices of the first and last samples in the time interval respectively. The wavelet power was integrated over frequency to calculate the power over frequency intervals, *P*_*j*(int)_, which in the discrete sense corresponds to

$$P_{j\left(int\right)}=\overset{f_{H}}{\underset{f_{k}=f_{L}}{\sum }}P_{j}\left(f_{k}\right),$$

where *f*_*L*_ and *f*_*H*_ are the lowest and highest frequencies in the interval respectively. The specific intervals used were 0.021–0.052 Hz, 0.052–0.145 Hz and 0.145–0.6 Hz, which were identified as physiologically-significant by previous studies (Stefanovska, 2007). These were chosen for two reasons: Firstly, they include frequencies corresponding to variability in the vascular resistance, which are important for the analysis of blood pressure variability and non-autonomic sources of cardiovascular regulation. The common alternative set of intervals chosen by the heart rate variability Task Force do not translate to multisignal analysis (Task Force of the European Society of Cardiology the North American Society of Pacing Electrophysiology, 1996). Secondly, these intervals were detected using similar methods with a logarithmic frequency scale (Stefanovska, 2007). Each band also has a defined minimum frequency, rather than the extremely ambiguous lower limit of 0 Hz provided by alternative definitions (Shaffer and Ginsberg, 2017).

The power curves of the sympathetic nerve activity signals were additionally summed over the 0.6–5 Hz interval due to the higher effective sampling frequency. In addition, the total power of fluctuations within a signal *x* for the time interval was calculated simply as

$$\text{Total power}=\frac{1}{n_{j}-n_{j}^{\prime }+1}\overset{n_{j}^{\prime }}{\underset{n=n_{j}}{\sum }}|x\left(n\right){|}^{2}.$$

Normalised frequency spectra were calculated by dividing the averaged wavelet power by the sum of the power across all frequency intervals. Interpretation of such normalised spectra requires care and can be a source of controversy (Eckberg, 1997). This is because the normalised power at specific frequencies can increase or decrease, while the actual power at these frequencies remains the same or even changes in the opposite direction after the same treatment. However, in this study the normalised power is useful to test the hypothesis that the blocking drugs have a stronger effect on the power at some frequency intervals relative to others, while the null hypothesis is that the blocking drugs act as an amplifier or attenuator (affecting all frequencies equally). Additionally, the normalised power spectrum has the advantage of showing changes independent of the baseline power spectrum of the individual.

#### 2.4.3 Determining the Presence of Shared Oscillations in Different Signals—Wavelet Phase Coherence

The wavelet transform was also used to compute the phases of the oscillations at each frequency within the signals. Using complex notation, *W*_*T*_(*s*, *t*) = *a*_*s*,*t*_ + *b*_*s*,*t*_ *i*, the corresponding phase was defined as *ϕ*(*s*, *t*) = tan^−1^(*b*_*s*,*t*_/*a*_*s*,*t*_). Considering the wavelet transforms of two signals, the difference in the phase at a specific time and frequency can be computed as

$$\text{Phase shift}=\Delta \phi_{s,t}=\phi_{1}\left(s,t\right)-\phi_{2}\left(s,t\right),$$

where *ϕ*_1_(*s*, *t*) and *ϕ*_2_(*s*, *t*) are the phases from each wavelet transform. The average phase difference was computed in order to detect phase shifts between the oscillations in two simultaneously-recorded signals.

To measure the similarity of the oscillations in the signals, the phase coherence was then defined as (Le Van Quyen et al., 2001; Lachaux et al., 2002)

$$\text{Phase coherence}=\sqrt{\langle \cos\Delta \phi_{s,t}\rangle^{2}+\langle \sin\Delta \phi_{s,t}\rangle^{2}}.$$

The phase coherence takes a value between 0 (no coherence) and 1 (coherence) (Bandrivskyy et al., 2004).

To determine the significance of intermediate values and also to negate the inherent bias towards lower frequencies, iterated amplitude-adjusted Fourier transform surrogates were adopted (Schreiber and Schmitz, 2000). These had the same spatial and frequency distribution as the signals under analysis but were otherwise uncorrelated. In each case the phase coherence between 100 pairs of surrogates was calculated and the 95th largest value of the distribution (i.e., the 95% level) was used to identify significant coherence in the actual signals. By definition, all linear effects are included in the surrogate data series, which means any significant effects above the surrogate level are nonlinear in origin. As such, changes in the linear characteristics of the dynamics due to experimental intervention do not influence this significance.

One issue with surrogate time series is that nonstationarity is not preserved, which means that low-frequency spikes or trends in the data can influence the assessment of significance (Schreiber and Schmitz, 2000). Here, we have mitigated these effects by detrending the signals and by measuring the subjects during a resting state.

#### 2.4.4 Determining the Influence of One Signal on Another—Conditional Mutual Information

Conditional mutual information (Paluš and Stefanovska, 2003), along with transfer entropy (Schreiber, 2000), belongs to a group of methods based on information theory. It characterises the complexity of a signal and estimates how much of this complexity originates from other input signals, thereby determining the strength and direction of the information transfer between the signals. When applied to biomedical data this can be used to identify couplings and their associated physiological functions (Jamšek et al., 2010).

In conditional mutual information a transfer of information is determined if the fluctuations in one signal influence the future fluctuations of another signal—a principle known as Granger causality (Vejmelka and Paluš, 2008). For Gaussian variables this quantity is equivalent to the transfer entropy used in other studies (Barnett et al., 2009). Granger causality has previously been applied in the study of cardiovascular regulation using both linear model-based approaches and nonlinear model-free approaches (Porta et al., 2013, 2014). These methods consider the observed variables to be a closed system and assume that there are no latent variables which can cause spurious detections of causality (Eichler, 2013; Porta et al., 2013). However, in the current study we focus on the decomposition of interactions between individual oscillatory modes, rather than analysing the flow of information in a fully-observed, closed system.

In addition, the method differs from dynamical Bayesian inference methods, which allow the complete coupling functions between oscillatory modes to be investigated (Iatsenko et al., 2013). In this case we selected a simpler method which provides details of the strength and direction of causal couplings without the need to specify a model (Clemson and Stefanovska, 2014). This is suitable for the current application where the direction of couplings (rather than the coupling functions) are the primary concern.

The conditional mutual information was calculated using the general approach from Paluš and Stefanovska (2003). The only difference was that due to the broadband frequency spectra of the measured signals, phases for the underlying oscillations could not be extracted. As such, the analysis used the original signals rather than time series of the extracted phases.

For each pair of signals the amplitudes were first normalised by subtracting the mean and dividing by the maximum absolute value. The data from each signal was sorted into four bins equidistantly-spaced between the minimum and maximum value. The 1-dimensional probability mass functions *P*(*x*_*j*_) were found by calculating the number of samples falling within each bin and dividing this number by the total number of samples. The 2-dimensional probability mass functions *P*(*x*_1_, *x*_2_) were generated in the same fashion from a corresponding set of 16 bins for the pairs of values sampled at the same times from the two signals. From these, the Shannon entropies were calculated using

$$H=-\overset{K}{\underset{k=1}{\sum }}P_{k}\ln\left(P_{k}\right),$$

where *P*_*k*_ refers to the *k*-th bin of the probability mass function *P* and *K* is the total number of bins. In the two-dimensional probability case, this procedure results in the *conditional* entropy *H*(*x*_1_|*x*_2_). The difference between the original signal and a delayed version of the signal was defined as Δ_*τ*_ *x*_*j*_(*n*) = *x*_*j*_(*n* + *τ*) − *x*_*j*_(*n*). The samples in these difference signals were also sorted into four equidistantly-spaced bins and the Shannon entropies were calculated in the same way as above for *H*(Δ_*τ*_ *x*_1_|*x*_1_), *H*(Δ_*τ*_ *x*_2_|*x*_2_), *H*(*x*_1_, Δ_*τ*_ *x*_2_|*x*_2_) and *H*(*x*_2_, Δ_*τ*_ *x*_1_|*x*_1_). In the latter two cases the entropies were calculated from 3-dimensional probability mass functions with a total of 64 bins. The conditional mutual information in each direction was then defined as (Paluš and Stefanovska, 2003)

$$I\left(x_{1};\Delta_{\tau }x_{2}|x_{2}\right)=H\left(x_{1}|x_{2}\right)+H\left(\Delta_{\tau }x_{2}|x_{2}\right)+H\left(x_{1},\Delta_{\tau }x_{2}|x_{2}\right),$$

$$I\left(x_{2};\Delta_{\tau }x_{1}|x_{1}\right)=H\left(x_{2}|x_{1}\right)+H\left(\Delta_{\tau }x_{1}|x_{2}\right)+H\left(x_{2},\Delta_{\tau }x_{1}|x_{1}\right).$$

In this case, *I*(*x*_1_; Δ_*τ*_ *x*_2_|*x*_2_) determines the influence of *x*_1_ on the future fluctuations of *x*_2_, while *I*(*x*_2_; Δ_*τ*_ *x*_1_|*x*_1_) gives the information flow in the opposite direction. The value of the conditional mutual information also depends on the parameter *τ* so the mean conditional mutual information over the range 0.5 s <τ< 50 s was used in the analysis of each 5 min interval to determine the degree of coupling between two signals.

In a similar way to phase coherence, even two completely independent noise signals can appear to have some information transfer. The same surrogate procedure described above was therefore applied to determine significant conditional mutual information. In addition, to separate the interactions occurring in difference frequency intervals, the signals were first processed using two bandpass finite impulse response filters for the intervals 0.052–0.145 Hz and 0.145–0.6 Hz.

### 2.5 Statistics

A repeated measures scheme was adopted to look for significant changes in the time averaged values and wavelet power of the signals for each treatment of the protocol. Given the small sample size, we could not confidently assume a normal distribution of the means for each treatment. The Friedman analysis of variance by ranks was therefore used as a non-parametric test of significance, given by *p* < 0.05. Tukey’s honest significant difference criterion was used to account for the multiple comparisons made for the data generated by each analytical method. In the case where the results were compared from both the spontaneous breathing and fixed-frequency breathing data, the two sets of data were included as separate rows in the same multiple comparisons test.

In the case of the phase coherence and conditional mutual information, the Wilcoxon signed-rank test was applied to the paired data of the calculated values and the 95% level from the surrogates. As in the other tests, significant interactions were defined as those with *p* < 0.05. The sum of the number of significant interactions for the specific treatments was calculated for each subject. The Friedman analysis of variance was then applied to these values to test the null hypothesis that the number of significant interactions is the same against the alternative hypothesis that the number of significant interactions changed between the treatments.

## 3 Results

### 3.1 Time-Averaged Analysis

As expected, atropine significantly reduced the mean R-R interval and increased mean systolic and diastolic blood pressure values (Table 1). Total power in the R-R interval, systolic pressure and diastolic pressure signals also appeared to be reduced. In contrast, the increase in R-R interval after propranolol was not found to be significant but the mean breathing rate did lower significantly when compared to saline. It can also be seen that the values for the double atropine-propranolol blockade follow those for atropine more closely than the values for propranolol.

**TABLE 1:** Time-averaged data for each of the 5 treatments during spontaneous breathing.

	Saline 1	Saline 2	Atropine	Propranolol	Double
Mean heart rate, Hz	1.03	0.93	1.75*^,^^†^	0.94	1.51^†^
	(+0.17,−0.22)	(+0.23,−0.01)	(+0.18,−0.13)	(+0.11,−0.08)	(+0.11,−0.06)
Mean breathing rate, Hz	0.21	0.21	0.23^†^	0.19*	0.21
	(+0.02,−0.03)	(+0.04,−0.02)	(+0.00,−0.03)	(+0.01,−0.04)	(+0.01,−0.01)
Mean systolic pressure, mmHg	129.5	132.7	153.7*	146.8	158.6
	(+0.4,−3.9)	(+12.1,−7.8)	(+21.5,−12.8)	(+12.9,−8.0)	(+13.0,−28.5)
Mean diastolic pressure, mmHg	76.1	78.4	93.8^†^	84.3	91.6
	(+4.8,−7.0)	(+4.1,−11.0)	(+9.9,−12.0)	(+9.9,−2.7)	(+17.2,−9.8)
R-R interval total power, ms^2^	2153	2241	14^†^	4,647	11*^,^^†^
	(+5,399,−506)	(+5,774,−551)	(+14,−6)	(+9,981,−1,671)	(+7,−7)
Systolic total power, mmHg^2^	15.1	20.1	4.9	9.8	6.6
	(+2.8,−6.3)	(+6.9,−8.4)	(+7.5,-0.7)	(+17.2,−1.9)	(+11.6,−1.0)
Diastolic total power, mmHg^2^	5.8	7.6	2.4	9.2	4.9
	(+3.0,−1.6)	(+6.2,−3.7)	(+1.4,−0.3)	(+4.0,−5.7)	(+1.9,−2.8)
Sympathetic activity total power (AU^2^)	29.3	17.0	21.9	22.7	11.2
	(+40.2,−17.2)	(+30.4,−2.0)	(+17.4,−17.0)	(+16.9,−3.7)	(+27.7,-5.8)
Respiration total power, %^2^	4.01	3.79	3.91	3.76	3.18
	(+0.57,−0.94)	(+1.12,−0.27)	(+0.39,−1.16)	(+0.99,−1.11)	(+1.16,−2.29)

The values shown are “median (75th percentile, 25th percentile)” over the 7 subjects. For the atropine and double treatments, significant (*p* < 0.05) differences with respect to saline 1 are marked by *, while significant differences with respect to propranolol are marked by ^†^.

### 3.2 Wavelet Power

The durations of the measurements within the apnea data were shorter than the fixed 5-min intervals (see Supplementary Material S1), which meant the power in the 0.021–0.052 Hz range could not be accurately determined. Additionally, the shortness of the apnea data meant that the phase coherence and conditional mutual information could not be calculated.

**FIGURE 1:**
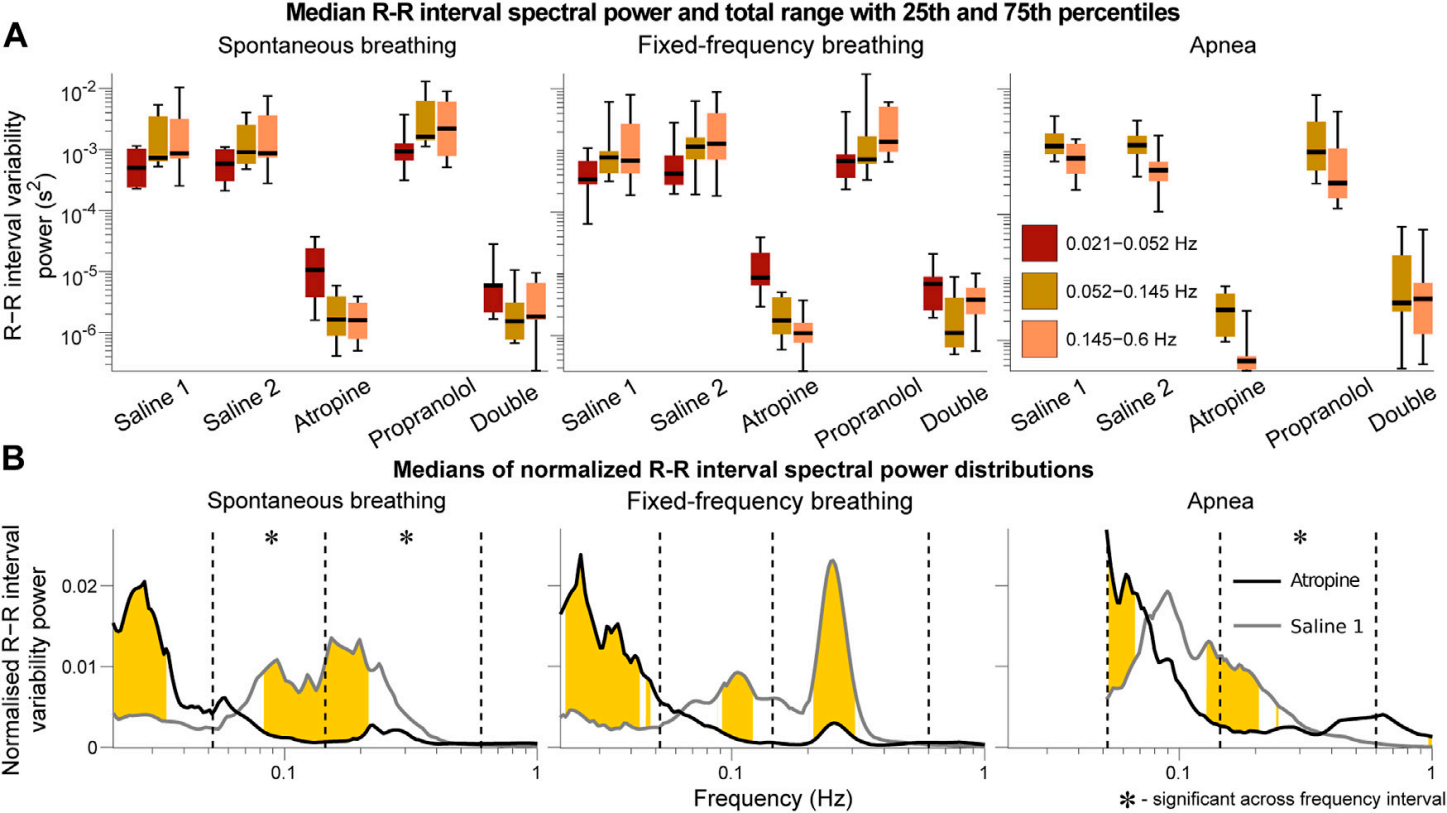
Spectral analysis of the R-R interval signals*.* The power in each frequency interval was found by time-averaging over the corresponding section of the wavelet transform. In **(A)** the median values are given by the black horizontal lines which intersect the colored boxes. The top and bottom of the boxes are located at the 75th and 25th percentiles respectively and the whiskers give the total range. In **(B)** the power distributions were normalised by dividing by the total power (sum of the curve) to compare the relative changes in the frequency distributions. The black and grey lines are the medians across all of the subjects. Data from the 0.021–0.052 Hz interval could not be determined for the apnea results due to the shortness of the measurements. Frequencies where the null hypothesis of the Friedman test was rejected for the saline 1 and atropine results are shown in yellow. The results indicate that mechanisms other than those related to baro-afferent activity are responsible for R-R interval variability in the 0.145–0.6 Hz frequency interval.

**FIGURE 2:**
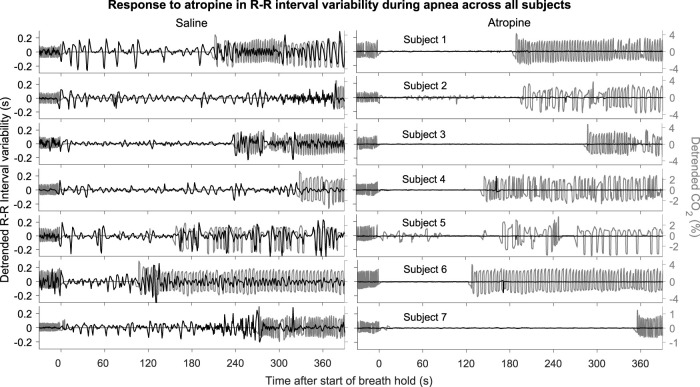
R-R intervals during apnea*.* The plots show the detrended R-R interval (black lines) and CO_2_ signals (grey lines) for each subject. The signals are shown from 30 s before to 390 s after the start of apnea.

The power across all frequency intervals in the R-R interval variability can be seen to strongly diminish after atropine (Figure 1A). Most importantly though, this is even true for the 0.145–0.6 Hz “respiratory frequency” interval during apnea. The corresponding oscillations that occur during apnea and are abolished by atropine are shown in the time domain in Figure 2.

The graphs of the normalised power (Figure 1B) also show that there is a shift in spectral distributions from the 0.145–0.6 Hz and 0.052–0.145 Hz intervals to the 0.021–0.052 Hz interval after atropine. The tiny size of the fluctuations after atropine might make this result seem negligible but it is still statistically significant. In all other cases most of the power is in the 0.145–0.6 Hz and 0.052–0.145 Hz intervals while after atropine most of the power is in the 0.021–0.052 Hz interval. This indicates that atropine had a greater effect in reducing the power across the higher-frequency intervals compared to the 0.021–0.052 Hz interval.

The same analyses were also performed using the frequency intervals specified by the Task Force on heart rate variability (Task Force of the European Society of Cardiology the North American Society of Pacing Electrophysiology, 1996). The results provided in the Supplementary Material S1 show the same effects described above, with the main difference being the significance over the frequency intervals of the normalied power. However, since a change in the normalised power of one interval necessarily indicates a shift of this power to one or more of the other intervals, the fact that the significance over specific intervals is different does not change the interpretation.

### 3.3 Phase Coherence and Conditional Mutual Information

**FIGURE 3:**
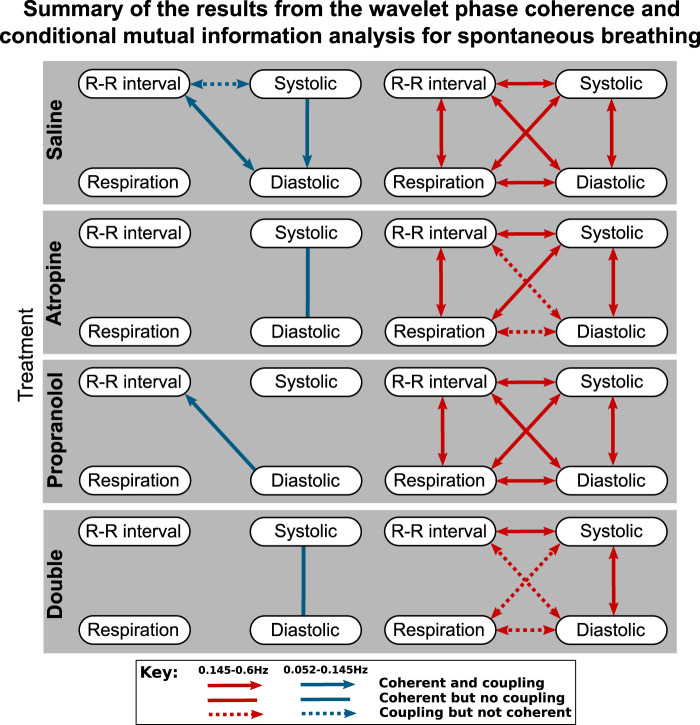
Diagrams showing the interactions detected for spontaneous breathing during each of the treatments*.* The significant couplings and coherences were found using conditional mutual information and wavelet phase coherence respectively. The results of the two saline treatments have been combined so that only the interactions common to both are shown. Interactions with the sympathetic nerve activity signals are shown separately in the Supplementary Material S1.

To simplify the interpretation, a summary of the results from the coupling and coherence analysis have been combined in Figure 3. While most of the significant interactions were common in the two saline measurements, there were also some differences. A significant interaction in the combined results was therefore defined as when the Wilcoxon signed-rank test gave *p* < 0.05 in one of the saline controls and at least *p* < 0.1 in the other control measurement.

One consideration to note when reviewing these results is that if there is coherence between two pairs of signals A–B and B–C then coherence is not necessarily found in A–C as the coherence can be generated by different fluctuations in the same frequency interval.

**FIGURE 4:**
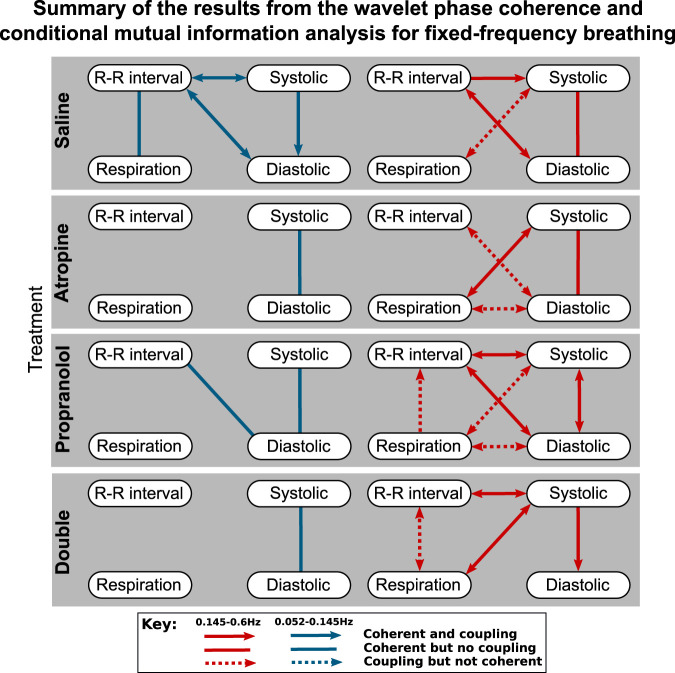
Diagrams showing the interactions detected for fixed-frequency breathing during each of the treatments*.* The significant couplings and coherences were found using conditional mutual information and wavelet phase coherence respectively. The results of the two saline treatments have been combined so that only the interactions common to both are shown. Note that during fixed-frequency breathing the dynamics of the respiration signals consist of a stationary oscillation. This means that significant interactions in the 0.145–0.6 Hz interval are difficult to define as the same oscillations are present in the surrogate data they are tested against.

The results of the statistical hypothesis tests showed a significant decrease in the number of interactions after both atropine and the double blockade when compared with the saline measurements. No significant difference was seen in the number of interactions after propranolol or when comparing the spontaneous and fixed-frequency breathing measurements (Figure 4) for the same treatments.

For spontaneous breathing, the interactions from the saline controls in the 0.145–0.6 Hz interval include strong bidirectional couplings and coherence between all of the signals. This adds support to the findings found in previous studies (Porta et al., 2013). For the 0.052–0.145 Hz interval the interactions do not appear to be as strong, with only one having significant bidirectional coupling and coherence.

In contrast to the results for the diastolic pressure, the systolic pressure signal remains generally coherent with the R-R intervals in the 0.145–0.6 Hz range after atropine. These results are shown in more detail in the Supplementary Material S1, where it can be seen that the coherence in the 0.052–0.145 Hz interval is slightly less significant after propranolol, while atropine removes all significant coherence across the frequency interval.

The effect of propranolol appears to be more subtle. None of the interactions in the 0.145–0.6 Hz interval are removed, although in the 0.052–0.145 Hz interval it does remove some of the interactions with the systolic pressure. In Figure 3 there appears to be some effect of propranolol when combined with atropine as there are fewer interactions after the double blockade when compared with atropine. However, this was not found to be significant in the statistical hypothesis test.

The responses were also different in spontaneous and fixed-frequency breathing, where in fixed-frequency breathing some of the interactions between respiration and the other signals were lost. However, the difference in the total number of interactions was not found to be significant.

### 3.4 Phase Shift

**FIGURE 5:**
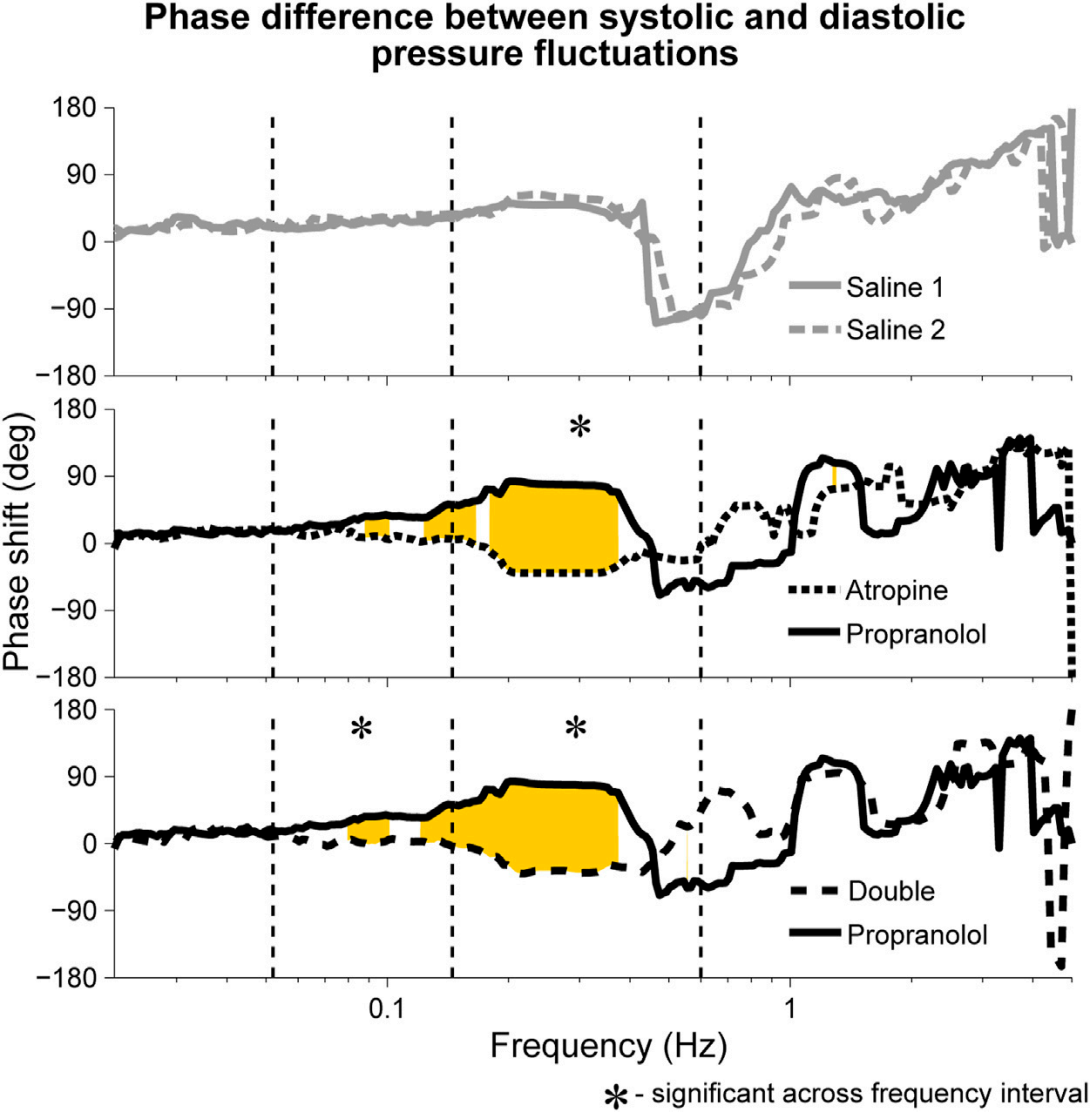
Median phase shift in the wavelet phases of systolic and diastolic blood pressure oscillations during fixed-frequency breathing. The shifts are shown after atropine, propranolol and the double blockade as well as for the two saline controls. Similar phase shifts were seen during spontaneous breathing, with the main difference being that the shift was not found to be significant across the 0.145–0.6 Hz interval but was significant across the 0.052–0.145 Hz interval for both atropine and the double blockade vs. propranolol. Rejection of the null hypothesis of the Friedman test, indicating significant phase shifts, are shown in yellow.

**FIGURE 6:**
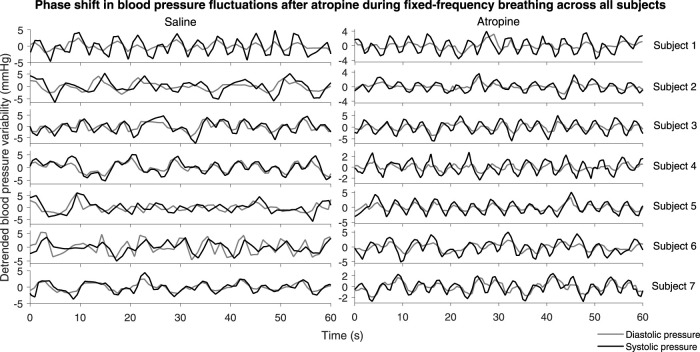
Systolic and diastolic pressure during fixed-frequency breathing*.* The plots show the detrended systolic (black lines) and diastolic (grey lines) blood pressure signals for each subject.

A significant difference in the phase shift was found in the systolic-diastolic pressure coherence in both the 0.052–0.145 Hz and 0.145–0.6 Hz intervals (Figure 5). For both of the saline controls the oscillations at these frequencies are still coherent, meaning changes in phase and frequency are the same, but not in-phase. The phase shift is larger for higher frequencies, suggesting that there is in fact a common time-lag across the frequency intervals. The positive value of the shift indicates that the oscillations in the diastolic pressure are leading the ones in the systolic. In contrast, after atropine the shift is removed in the 0.052–0.145 Hz interval, meaning the oscillations are in phase and rise and fall together. In the 0.145–0.6 Hz interval the phase shift actually becomes slightly negative, meaning the respiratory oscillation in the systolic pressure is leading the oscillation in the diatolic pressure. The effect can also be seen in the time domain time as shown in Figure 6. In these plots the fluctations in the diastolic pressure can be seen to precede those in the sytsolic pressure after saline. However, after atropine the fluctuations in the systolic pressure are in phase with or slightly precede those in the diastolic pressure.

In addition, a significant phase shift was also observed in the 0.145–0.6 Hz interval for the phase coherence between respiration and systolic pressure (see Supplementary Material S1), indicating that after atropine the respiratory oscillation in the systolic pressure was falling further behind the actual respiration. In contrast, no positive phase shift in the phase coherence between respiration and diastolic pressure was observed after atropine. However, this was likely due to the fact that the coherence of the respiratory oscillation in the diastolic pressure signals was not found to be significant.

Although not significant, it can be seen in Figure 5 that the median phase shift for propranolol appears larger than the phase shift observed in the saline controls. It is therefore possible that propranolol had the opposite effect to atropine, creating a positive shift in phase as opposed to a negative one.

## 4 Discussion

The results have revealed two major new findings: 1. R-R interval variability in the 0.145–0.6 Hz range that persists during apnea is removed after atropine, which has implications for the mechanism that produces respiratory sinus arrhythmia. 2. There is a change in phase shift between systolic and diastolic pressure fluctuations after autonomic regulation is blocked, which is measurable due to the existence of non-autonomic mechanisms.

The first finding suggests that calculations of baroreflex sensitivity based on variability that includes the respiratory sinus arrhythmia are unreliable. Furthermore, the second finding provides a new way of measuring autonomic cardiovascular regulation, as an alternative to the baroreflex sensitivity. These points are expanded further in the discussion below.

In addition to these implications for measurements of baroreflex sensitivity, the results also suggest a parasympathetic-modulated mechanism contributes to the increase in sympathetic activity observed during apnea. They also reveal potential methodological problems in cases where fixed-frequency breathing is used.

### 4.1 Respiratory Sinus Arrhythmia Is Centrally-Mediated

Previous studies have proposed that the mechanism behind the generation of respiratory sinus arrhythmia is purely reflex-based (Piepoli et al., 1997; Karemaker, 2009). However, this theory is inconsistent with the results found for R-R interval variability during apnea. The hypothesis of the previous studies implies that there should be no respiratory-frequency oscillations in the R-R interval variability due to the lack of baro-afferent activity generated by the breathing cycle. The results of saline measurements during apnea show clear respiratory-frequency oscillations, which provides opposing evidence for this hypothesis. An alternative hypothesis is that these oscillations are post-synaptic in origin. However, this would mean that there should be no difference in the R-R interval variability at the breathing frequency between the saline control and during either of the pharmacological blockades. Instead, the results presented here show that there is still a huge reduction in R-R interval variability in this frequency range after atropine. The remaining hypothesis is that the oscillations are pre-synaptic in origin but are not generated *via* the baroreflex.

The persistence of variability in the same frequency range as respiratory sinus arrhythmia during apnea has also been observed previously in dogs (Horner et al., 1995). This suggests that a significant amount of this variability originates from a central mechanism in the brainstem (Berntson et al., 1993; Elstad et al., 2018). This adds to other evidence which shows that the computation of baroreflex sensitivity based on the measurement of respiratory sinus arrhythmia is unreliable (Badra et al., 2001; Carrasco-Sosa et al., 2005; Tiinanen et al., 2008).

### 4.2 Evidence for Non-Autonomic Modulation of the Cardiovascular System

Several results point to a significant non-autonomic influence on the variability in the cardiovascular system. Relative to the other frequency intervals, the dynamics of the R-R interval variability in the 0.021–0.052 Hz range is more resistant to the decrease in power caused by a parasympathetic blocker and is also unaffected by a sympathetic blocker. If these oscillations were generated by mainly pre-synaptic mechanisms like those in the other frequency intervals then we would expect a similar decrease in power. This is despite the fact that baroreflex sensitivity fluctuations have been observed to be concentrated in this range rather than at the higher frequency intervals (Eckberg and Kuusela, 2005). However, this gives support to other studies which have shown that the oscillations at this frequency may be influenced by post-synaptic heart rate modulation related to thermoregulation (Fleisher et al., 1996; Sollers et al., 2002). Another study has shown that these temperature-related changes can be induced through visual information (Takakura et al., 2013). Certainly, these very low frequency oscillations are mainly influenced by autonomic-dependent mechanisms that include the parasympathetic system (Taylor et al., 1998), the renin-angiotensin system (Duprez et al., 1995), anti-baroreflex functions and chemoreceptor activity (Ponikowski et al., 1997; Francis et al., 2000). Understanding the exact cause of the oscillations that remain after autonomic blockade is therefore still up for debate. It should also be noted that the stronger effect of atropine on the other frequency intervals should not mean that they reflect proportional changes in parasympathetic activity, which has been shown in previous studies (Kollai and Mizsei, 1990).

The results of the coupling and coherence analysis also provide evidence of non-autonomic interactions. In particular, the persistence of the R-R interval–systolic pressure interaction in the 0.145–0.6 Hz frequency interval after both blocking drugs also suggests that there may be a non-autonomic mechanism involved in its generation. This could be related the mechanical influence of the respiration on the R-R interval *via* the pressure applied on the sinus node (Swenne, 2013).

### 4.3 Non-Autonomic Influence on Blood Pressure Variability Explains the Systolic–Diastolic Phase Shift After Autonomic Blockade

Indications of the influence of non-autonomic mechanisms on the cardiovascular system can also been seen in the differences between the systolic and diastolic blood pressure variability. While general blood pressure variability has been observed since the 18th century [see (Billman, 2011) and the references therein], very little work has been done to investigate these differences. However, a change in the phase shift in the systolic-diastolic blood pressure oscillations after atropine was observed by Triedman and Saul in a paper from 1994 (Triedman and Saul, 1994). In that work they measured the coherence between central venous pressure fluctuations and blood pressure variability using frequency domain analysis. They showed that a change in venous pressure was reflected in the systolic pressure after a time lag of 1.55 s and in the diastolic pressure after a time lag of 2.1 s. The phase shifts between venous pressure and the systolic and diastolic pressure variations were shown to equalise across much of the frequency domain after autonomic blockade. Furthermore, they noted that the phase relationship between the instantaneous lung volume and the diastolic pressure variations changed by 90° after autonomic blockade, causing it to match the relation between lung volume and systolic pressure variations.

The explanation that the authors give for the change in phase shift is that during autonomic blockade the blood pressure variations reflect the mechanical effects of intrathoracic pressure on arterial pressure. In contrast, under control conditions a different phase relation is seen between respiration and blood pressure oscillations because the heart-respiration interaction is the predominant modulator.

The results presented here in Figure 5 confirm the findings of Triedman and Saul. The phase shift in the respiration-systolic pressure phase coherence suggest that it is in fact the systolic rather than the diastolic pressure variations which change in phase, although a less-pronounced phase shift in systolic variability was also reported by Triedman and Saul (1994). However, the significant phase shifts below the respiratory frequency range do not align with the assumption that the blood pressure variability after autonomic blockade originates from intrathoracic pressure changes. In Triedman and Saul’s paper, this was explained by the experimental protocol where the respiratory frequency distribution was artificially widened using random-interval breathing. This explanation does not hold for the current results though, where significant phase shifts in the lower frequency interval were seen during fixed-frequency breathing. Rather, this suggests there are other sources of non-autonomic blood pressure variability.

### 4.4 Sources of Non-Autonomic Influences in Blood Pressure Variability

Evidence for the modulation of blood pressure variability by a mechanism other than autonomic effects has been observed in rats, where the variations in systolic blood pressure were attributed to changes in stroke volume (Japundzic et al., 1990). A subsequent study with human subjects found these respiration-synchronous fluctuations to be inversely proportional to those in the R-R interval variability (Toska and Eriksen, 1993). The superposition of these two sources of fluctuations actually reduces the cardiac output variation under control conditions. It was observed that when the R-R interval fluctuations are blocked by atropine this results in an increase in fluctuations of cardiac output and arterial pressure. An alternative explanation for this effect was proposed by Taylor and Eckberg (1996), who observed reduced arterial pressure fluctuations during fixed-rate atrial pacing. They suggested that altered arterial compliance caused by the blocking drugs is the reason for the increased blood pressure variability seen in some of the earlier studies.

Neither of these explanations fully describe the results seen here. Indeed, comparing Figure 1 and the spectral analysis of the systolic blood pressure signals (provided in the Supplementary Material S1) reveals little significant change in the blood pressure variability after atropine despite a huge reduction in the R-R interval variability, with the most striking difference in the 0.145–0.6 Hz interval. This seems to confirm that the blood pressure variability is at least partly-generated by non-autonomic mechanisms. However, given that the two sources of variability indicated by Figure 5 are out of phase by <90° , the principle of wave superposition means that the power should decrease when the heart-respiration interaction is removed. This cannot simply be the effect of intrathoracic pressure changes, the magnitude of which should be correlated with the respiration total power which did not change significantly between the treatments (Table 1). Furthermore, the increased arterial compliance cannot be the only mechanism responsible for maintaining the power of the blood pressure variability since it *does* decrease in the 0.052–0.145 Hz interval, which is significant during both spontaneous breathing and apnea.

Instead, a possible candidate for the effect seen is a change in the myogenic response. Coherent oscillations related to myogenic activity have been shown to be ubiquitous throughout the cardiovascular system (Stefanovska and Hožič, 2000). Myogenic activity has previously only been found to generate oscillations in the 0.052–0.145 Hz range (Kvernmo et al., 1998), although the adjustment of myogenic activity to the respiration cycle has also been illustrated (Stefanovska et al., 2001). It is therefore possible that the actual effect being observed is an increase in the frequency of these myogenic oscillations, brought about by the change in the blood flow dynamics. If the myogenic oscillations were to move sufficiently close to the 0.145 Hz boundary, as hypothesised to occur under certain conditions in (Stefanovska et al., 2001), then some of their power would contribute to the 0.145–0.6 Hz interval. This would explain the reduction of the power in the 0.052–0.145 Hz interval.

### 4.5 The Phase Shift Between Systolic and Diastolic Pressure Fluctuations as a Marker of Autonomic Regulation

Besides the physiological implications, the phase shift in Figure 5 appears to act as a marker for functioning autonomic regulation of the cardiovascular system. It has already been shown that the phase relationship between heart rate and systolic pressure variations changes with ageing (Milan-Mattos et al., 2018)). However, Triedman and Saul (Triedman and Saul, 1994) only compared the phase shifts in the coherence of systolic and diastolic pressure variability relative to the central venous pressure and instantaneous lung volume. Here we have shown that there is in fact a change in the direct phase relation between the systolic and diastolic pressure variability. It is also interesting to note that, even without autonomic blockade, on timescales of 30 min and longer the systolic and diastolic blood pressure variations are strongly correlated (Mancia et al., 1983). This may therefore help to identify the maximum timescale of the autonomic regulation.

### 4.6 Parasympathetic Modulation of Sympathetic Activity in Apnea

**FIGURE 7:**
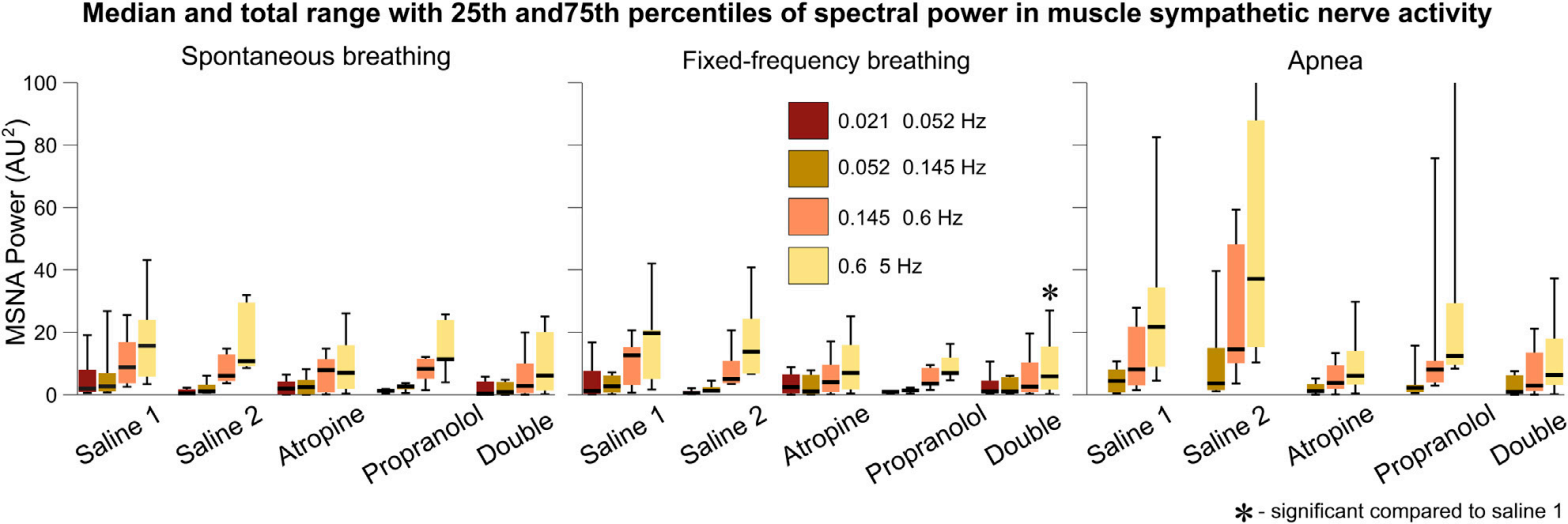
Spectral analysis of the sympathetic nerve activity signals. The power in each frequency interval was found by time-averaging over the corresponding section of the wavelet transform. The median values are given by the black horizontal lines which intersect the colored boxes. The top and bottom of the boxes are located at the 75th and 25th percentiles respectively. The whiskers give the total range, which extends beyond the axis limit in two cases shown for apnea.

Since atropine is a parasympathetic blocker it does not have a direct effect on the sympathetic nervous system. However, in the sympathetic nerve activity signals a slight decrease in the power of the 0.6–5 Hz interval can be seen after atropine, which is significant for the double blockade during fixed-frequency breathing (Figure 7). An increase in power across all frequency intervals can also be seen during apnea, as reported previously (Morgan et al., 1995). Atropine appears to reduce this effect, while the change in response after propranolol was a reduction in the median powers but with a wide range of responses from the different subjects.

This reveals an important physiological mechanism relating to the role of the parasympathetic system during apnea. The reduction in the power of the sympathetic nerve activity after atropine suggests that the sympathetic system is suppressed when the parasympathetic system is blocked. Such a feedback mechanism has been evidenced previously (Montano et al., 1998). Further study of the effect of atropine on the increase in sympathetic activity during apnea is needed to determine whether this effect is significant compared to any reduction in activity during spontaneous or fixed frequency breathing. It has previously been proposed that the increase in sympathetic activity power during apnea is related to stimulation of the carotid bodies from hypoxia or hypercapnia (Daly et al., 1979; Somers and Abboud, 1993). However, the fact that the subjects in the current study hyperventilated while breathing 100% oxygen for 2 min prior to the apnea measurements would suggest that this is not the cause. Instead, it is possible that a separate parasympathetic-modulated mechanism contributes to this increase in sympathetic activity. This seems consistent with the nonlinearity of the autonomic interactions within the cardiorespiratory system and the role of vagus nerve activity in obstructive sleep apnea (Parhizgar et al., 2011).

### 4.7 Methodology of Fixed-Frequency Breathing

The results highlight some important methodological implications. The reason for the apparent loss of significant interactions in the 0.145–0.6 Hz interval during fixed-frequency breathing is that the breathing cycle is an oscillation with a stationary frequency. This means that the dynamics of the Fourier transform surrogate time series in the 0.145–0.6 Hz interval are indistinguishable from the measured data. The only difference that remains is the effect of the interactions on the shape and amplitude of cycles of the tidal volume, which might not be as strong as the effect on the breathing rate in the conditional mutual information and do not influence the wavelet phase coherence. This effect is most surprising for the respiration to R-R interval coupling which characterises the respiratory sinus arrhythmia. This interaction has been explored in-depth in previous studies (Grossman and Taylor, 2007), and is seen in the R-R interval variability power in the 0.145–0.6 Hz interval (Figure 1). Since it is common to use fixed-frequency respiration as a way to limit the adverse effects that breathing has on other physiological parameters, this has important implications for the type of methods that should be used to determine the strength of the respiration - heart coupling. Other methods which are able to directly model and track the changes in the strength of interactions in time may be more suitable (Stankovski et al., 2012). It is also worth noting that experiments using ramped breathing do not face this problem even though the frequency is still externally paced (Stankovski et al., 2013).

The effects of fixed-frequency breathing data can also interact with the effects of the treatment and cause ambiguous results. This can be seen in the respiration-systolic pressure coherence, where it is only for atropine and the double blockade that the coherence is not lost during fixed-frequency breathing (Figure 3). This could perhaps be explained if the amplitude of the fixed-frequency respiration-related fluctations in the systolic pressure were reduced by the effect of atropine. This would increase the relative amplitude of any background fluctuations in the 0.145–0.6 Hz interval. Consequently, the surrogate data would no longer contain a single stationary oscillation and the coherence would appear significant again. However, no significant reduction in amplitude in the 0.145–0.6 Hz interval can be seen in the systolic blood pressure variability. One explanation might again be the mechanical impact that the respiration has on the cardiovascular system. The interaction is dominated by the autonomic baroreflex in healthy individuals, but when the baroreflex is diminished the mechanical effects can start to dominate instead (Swenne, 2013). Breathing also mechanically alters left ventricular stroke volumes (Ruskin et al., 1973). If this effect on the blood pressure occurs over a different time delay then the result is two oscillations around the same frequency superimposed on one another. This would give rise to phase variability in the surrogate data, which makes the underlying coherence appear significant.

### 4.8 Study Limitations

It is worth mentioning some of the limitations of the study to provide some more context for the results. The most obvious is the limited number of subjects which means the data is more strongly influenced by variations within individuals. This issue was tackled by using stricter non-parametric statistical hypothesis tests, using surrogate data and comparing against the saline controls at all times. While this may have reduced the likelihood of false positive results, it also means that some non-significant results may become significant if there were more subjects. However, the statistical power of the study was also improved by the large effect sizes seen for the different pharmacological blockades and as such these significant effects were still statistically observable.

The length of the data in time is another limitation of this study. It has already been mentioned that the shortness of the apnea data reduced the range of frequencies that could be observed, in addition to preventing the analysis of coherence and couplings. For the other 5 min long parts of the protocol these types of limitations are still relevant. For example, some of the responses to the blocking drugs may have not been observed because they occurred on timescales longer than 5 min. For low frequency oscillations that were in the observable range there are still fewer cycles of the oscillations, making it more difficult for changes at these frequencies to be statistically significant, causing a bias towards finding significance in results related to high-frequency dynamics. This might explain why the vast majority of the significant results correspond to changes after atropine, which affects the high-frequency vagal activity, while the changes after propranolol may be limited to the low-frequency ranges associated with sympathetic activity.

In the case of the time-frequency analysis, the choice of the frequency intervals can result in bias towards detecting some phenomena over others. To mitigate this, the analysis of the R-R interval variability was performed using two sets of intervals and no anomalous results were found.

A limitation of the observed changes in the power spectral distributions, both absolute and normalised, is that they are found by making comparisons across the whole group. This differs from the phase coherence and conditional mutual information results where surrogate data is used to account for the intra-individual variability. One possible way to achieve this when comparing spectral data would be to use inter-subject surrogate analysis (Toledo et al., 2002). However, this was not possible in the current study as it would require a larger number of subjects in order to capture the full range of variations in the spectral distributions of the control measurements.

Finally, the conditional mutual information-based method for finding the strength of interactions between two raw time series, while very powerful, does not differentiate between the various types of couplings. For example, the significant drop in conditional mutual information for interactions involving respiration during fixed-frequency breathing hints that the interactions observed during spontaneous breathing may be based on the modulation of the breathing rate rather than amplitude-based effects. The development of dynamical Bayesian inference of generalised coupling functions might be useful in this case (Stankovski et al., 2017).

### 4.9 Summary

Table 2 gives an overview of the physiological hypotheses that have been tested in the current study, along with the details of comparable studies that tested the same hypotheses. This work has made several contributions to the understanding of cardiorespiratory and cardiovascular interatctions, which we now summarise in the following list of findings:

• The results have revealed that respiratory sinus arrhythmia is centrally mediated and not generated solely by a reflex mechanism of the dynamic pressure changes caused by the breathing cycle.
• We have found new evidence for the modulation of the cardiovascular system by non-autonomic mechanisms. This non-autonomic influence explains a change in phase shift between systolic and diastolic pressure fluctuations. Furthermore, like the baroreflex sensitivity, this phase shift acts as a marker for autonomic regulation of the cardiovascular system and provides an alternative means of non-invasive assessment.
• A feedback loop exists that reduces sympathetic activity during apnea when parasympathetic activity is blocked. However, this does not affect sympathetic activity during either spontaneous or fixed-frequency breathing.
• Lastly, spurious results can arise from methods that test against surrogate data when fixed-frequency breathing is used. The effect of uncontrolled breathing amplitudes on frequencies below the fixed breathing rate should also be considered.

**TABLE 2:** Methods and results of the physiological hypotheses tested in the current study compared with those of previous studies.

Null hypothesis	Alternate hypothesis	References	Methods used	Result
Respiratory sinus arrhythmia (RSA) is generated by arterial pressure waves *via* the baroreflex	RSA is centrally-mediated	Current study	Wavelet power spectrum	Reduction in power in the respiratory frequency interval after parasympathetic blockage during apnea suggests variability in this interval is centrally mediated
		de Boer et al. (1987)	Fourier spectra and cross spectra, compared with simulated data from beat-to-beat model	The baroreflex model reproduced the same frequency spectra but only the case of spontaneous breathing was analysed
		Eckberg et al. (2016)	Time domain measures of R-R interval variability and baroreflex gain	Responses to apnea shown to be chemoreceptor-independent
		Simpson et al. (2019)	Time domain measures of baroreflex gain	Elimination of the peripheral chemoreceptor drive at high altitude did not influence the baroreflex measures, suggesting that other mechanisms control the vascular sympathetic baroreflex resetting
R-R interval and blood pressure variability is controlled entirely by sympathetic and parasympathetic nerve traffic	R-R interval and blood pressure variability is influenced by non-autonomic mechanisms	Current study	Wavelet power spectrum, wavelet phase coherence/shift, conditional mutual information	The persistence of the R-R interval-systolic pressure interaction at the respiratory frequency interval, persistence of R-R interval variability in the 0.021–0.052 Hz interval and phase shift of coherent oscillations in the systolic-diastolic variability support the influence of non-autonomic mechanisms
		Bernardi et al. (1989)	Spectra and cross spectra	Transplant patients with denervated hearts were found to have respiratory-synchronous peaks in the R-R interval variability spectra
		Porta et al. (2015)	Joint transfer entropy, self entropy and conditional self/joint transfer entropy	During head-down tilt RSA amplitude increased despite decreases in conditional information transfer from the respiration and systolic pressure signals, suggesting another physiological pathway is involved
The increase in sympathetic activity during apnea results from a chemoreceptor reflex in response to hypoxia and hypercapnia	Sympathetic activity is increased by a separate reflex mechanism that responds to an increase in parasympathetic activity during apnea	Current study	Wavelet power spectrum	Increase in sympathetic activity during apnea that appeared to be parasympathetic-modulated despite subjects beginning apnea in a state of hyperoxia and hypocapnia
		Somers et al. (1989)	Time domain analysis of MSNA signals	Found stronger sympathetic response to hypoxia compared with hypercapnia but did not investigate the case of hyperoxia with hypocapnia
Non-autonomic changes in blood pressure variability result entirely from thoracic pressure variability	The myogenic mechanism is able to generate non-autonomic variations in the central blood pressure variability	Current study	Wavelet power spectrum and wavelet phase coherence/shift	Observation of phase shift in systolic-diastolic variability coupled with no change in the power spectra suggests a controlling mechanism besides the autonomic control that maintains the magnitude of the blood pressure oscillations in the 0.145–0.6 Hz interval
		Triedman and Saul, (1994)	Fourier phase coherence/shift	Attributed phase shift in the blood pressure variability to the dominance of intrathoracic pressure after autonomic blockade, but did not compare changes in power spectra

## Data Availability

The datasets analysed for this study can be found in the Publications and Research (Pure) portal on Lancaster University’s research information management system, which at the time of publication they indicate is: https://dx.doi.org/10.17635/lancaster/researchdata/280.
