# Supplementary material for: Beyond the Baroreflex: A New Measure of Autonomic Regulation Based on the Time-Frequency Assessment of Variability, Phase Coherence and Couplings
Source: Front Netw Physiol. 2022 Jun 6;2:891604. doi: 10.3389/fnetp.2022.891604 (PMC10013010; doi:10.3389/fnetp.2022.891604)
Supplement: Supplementary file 1 [file DataSheet1.pdf]

Supplementary Material

1 APNEA DATA

Additional data from the apnea measurements is provided in Table S1.

2 BLOOD PRESSURE WAVELET POWER

The wavelet power results for the systolic blood pressure variability are shown in Figure S1. The results for the diastolic pressure variability followed a similar pattern.

3 SYMPATHETIC NERVE ACTIVITY INTERACTIONS

To simplify the interaction diagrams shown in main text, additional interactions relating to the sympathetic nerve activity are shown separately in Figure S2.

4 ADDITIONAL PHASE SHIFT RESULTS

Phase shifts results for all of the signal pairs and across all of the treatments are shown in Figure S3. In addtion, the other significant phase shift observed between respiration and systolic pressure is shown in more detail in Figure S4.

5 R-R INTERVAL WAVELET POWER USING DIFFERENT FREQUENCY INTERVALS

The results of the wavelet power analysis of R-R intervals using the frequencies specified by the heart rate variability Task Force are shown in Figure S5.

Table S1. *Time-averaged data for each of the 5 treatments during apnea.*

	Saline 1	Saline 2	Atropine	Propranolol	Double
Apnea duration, s	245<br>(+73,-72)	255<br>(+46,-13)	190<br>(+77,-38)	233<br>(+92,-22)	259<br>(+32,-92)
Mean heart rate, Hz	1.15<br>(+0.10,-0.25)	1.16<br>(+0.04,-0.13)	0.56*†<br>(+0.05, -0.05)	1.16<br>(+0.18,-0.08)	0.69†<br>(+0.04,-0.02)
Mean systolic pressure, mmHg	134.9<br>(+5.6,-6.4)	146.0<br>(+11.1,-8.0)	165.5<br>(+9.5,-25.3)	148.4<br>(+4.1,-25.6)	163.5*<br>(+2.6,-23.2)
Mean diastolic pressure, mmHg	89.8<br>(+4.9,-16.5)	96.3<br>(+9.7,-11.4)	98.6<br>(+21.2,-4.8)	87.6<br>(+16.2,-7.7)	99.0<br>(+22.4,-6.1)
R-R interval total power, ms^2	2348<br>(+723,-1064)	1807<br>(+588,-536)	4*†<br>(+3,-2)	1301<br>(+2813,-628)	17†<br>(+47,-11)
Systolic total power, $mmHg^2$	7.3<br>(+0.3,-1.4)	5.7<br>(+3.3,-1.9)	1.9*<br>(+2.1,-0.3)	5.8<br>(+1.6,-2.1)	2.3<br>(+2.0,-0.8)
Diastolic total power, $mmHg^2$	4.1<br>(+2.2,-1.7)	2.7<br>(+5.2,-1.3)	1.1*<br>(+1.1,-0.3)	4.2<br>(+0.5,-2.6)	1.1*<br>(+0.1,-0.1)
Sympathetic activity total power (AU^2)	34.1<br>(+33.5,-21.4)	55.3<br>(+86.2,-28.7)	14.9<br>(+13.9,-9.0)	25.5<br>(+16.7,-10.7)	10.6<br>(+31.4,-5.9)

The values shown are "median (75th percentile, 25th percentile)" over the 7 subjects. For the atropine and double treatments, significant ($p < 0.05$) differences with respect to saline 1 are marked by *, while significant differences with respect to propranolol are marked by †.

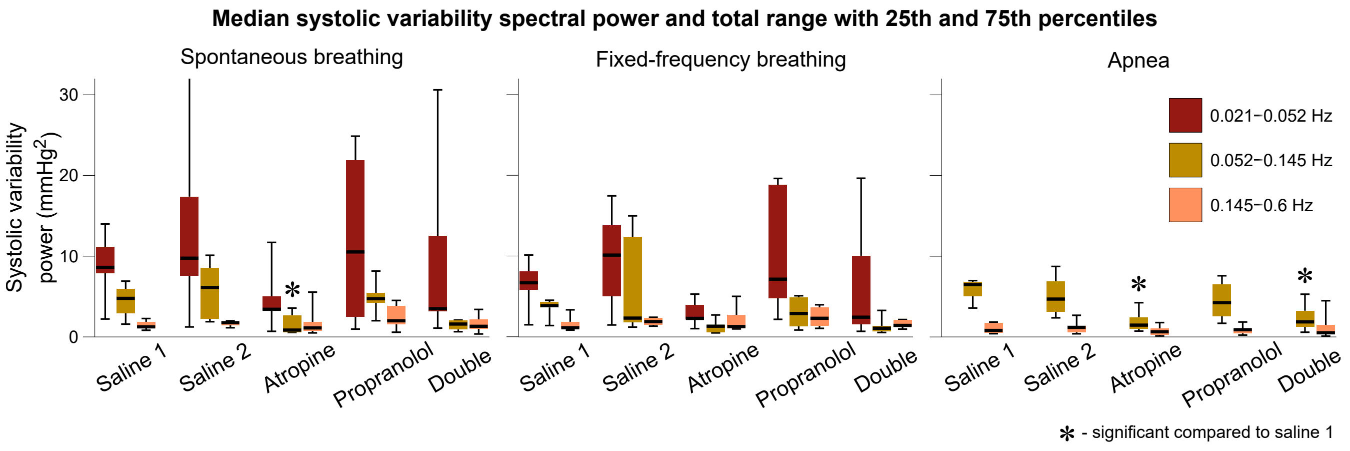

Figure S1. *Spectral analysis of the systolic blood pressure signals.* The power in each frequency interval was found by time-averaging over the corresponding section of the wavelet transform. The median values are given by the black horizontal lines which intersect the colored boxes. The top and bottom of the boxes are located at the 75th and 25th percentiles respectively. The whiskers give the total range, which extends beyond the axis limit in one of the results shown for spontaneous breathing. Significant changes were found between saline 1 and atropine as well as saline 1 and double (marked with *).

6 PHASE COHERENCE BETWEEN R-R INTERVALS AND SYSTOLIC BLOOD PRESSURE

The results of the wavelet phase coherence analysis for the R-R intervals and systolic blood pressure are shown in more detail in Figure S6.

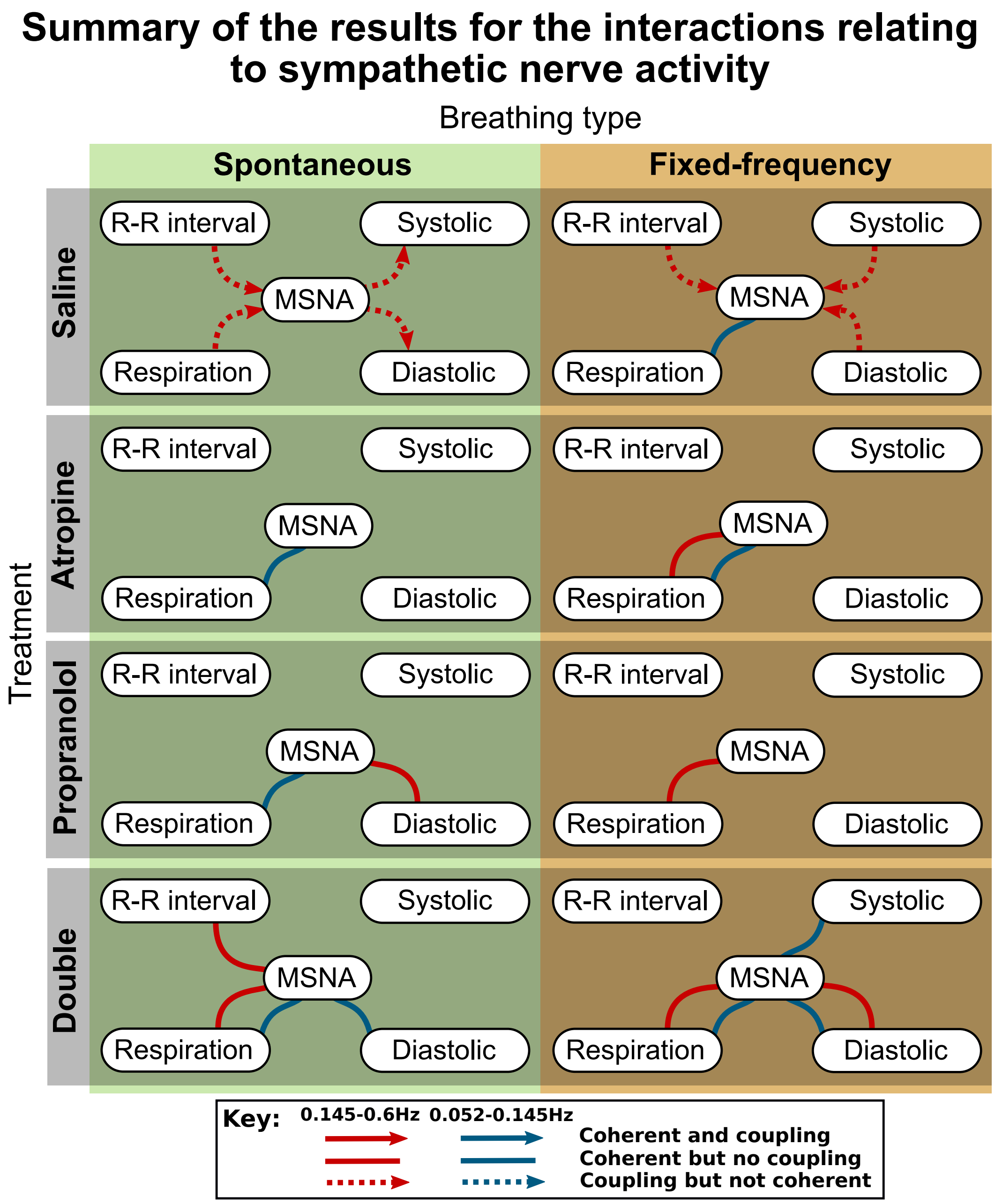

Figure S2. *Diagrams showing the interactions relating to the sympathetic nerve activity signals only.* The significant couplings and coherence were found using conditional mutual information and wavelet phase coherence respectively. The results of the two saline treatments have been combined so that only the interactions common to both are shown.

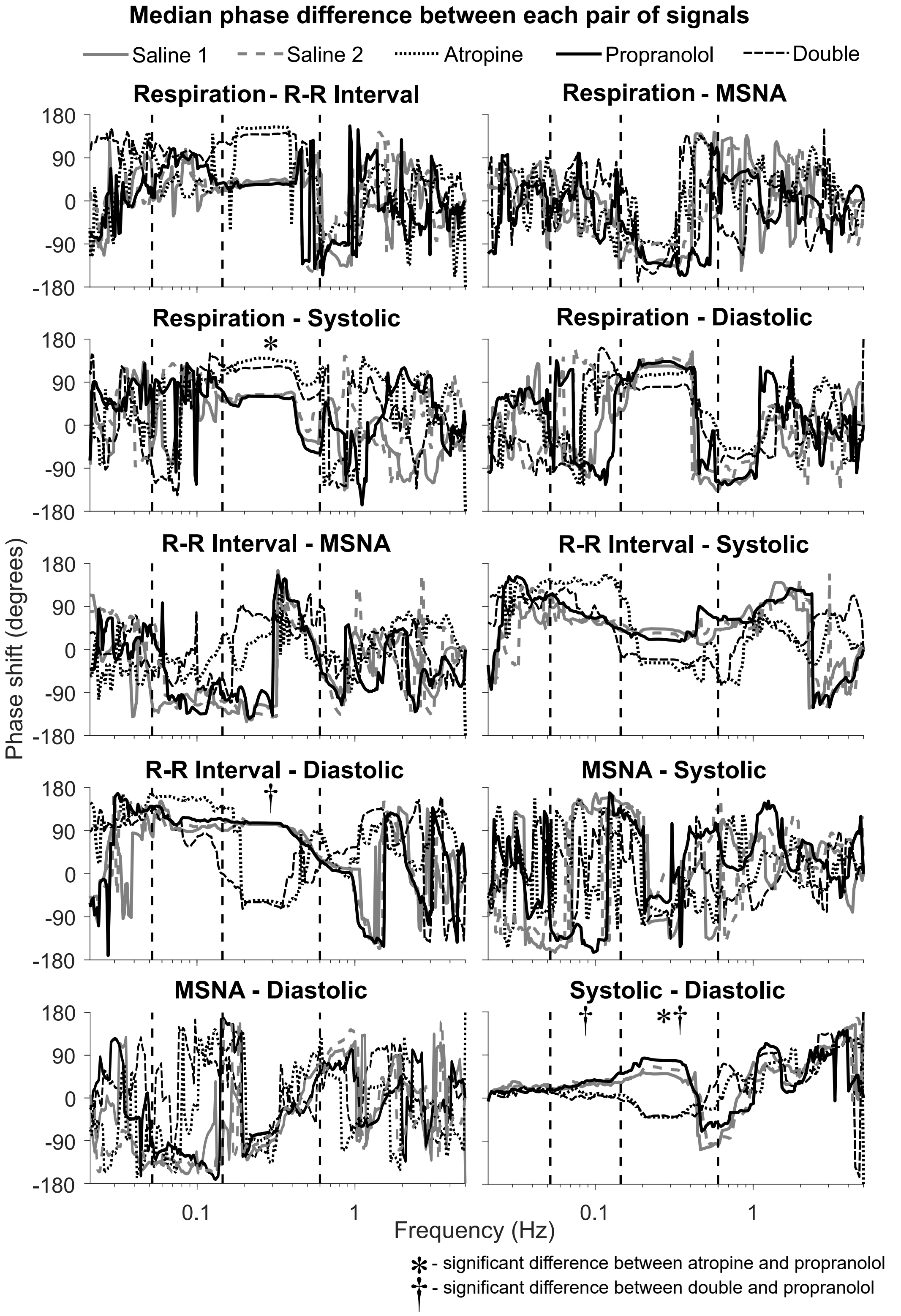

Figure S3. *Median phase shifts between all of the signal pairs in the case of fixed-frequency breathing.* The shifts are shown after atropine, propranolol and the double blockade as well as during the two saline controls. Markers indicate significance across the frequency interval, where * marks significance between atropine and propranolol and † marks significance between the double blockade and propranolol.

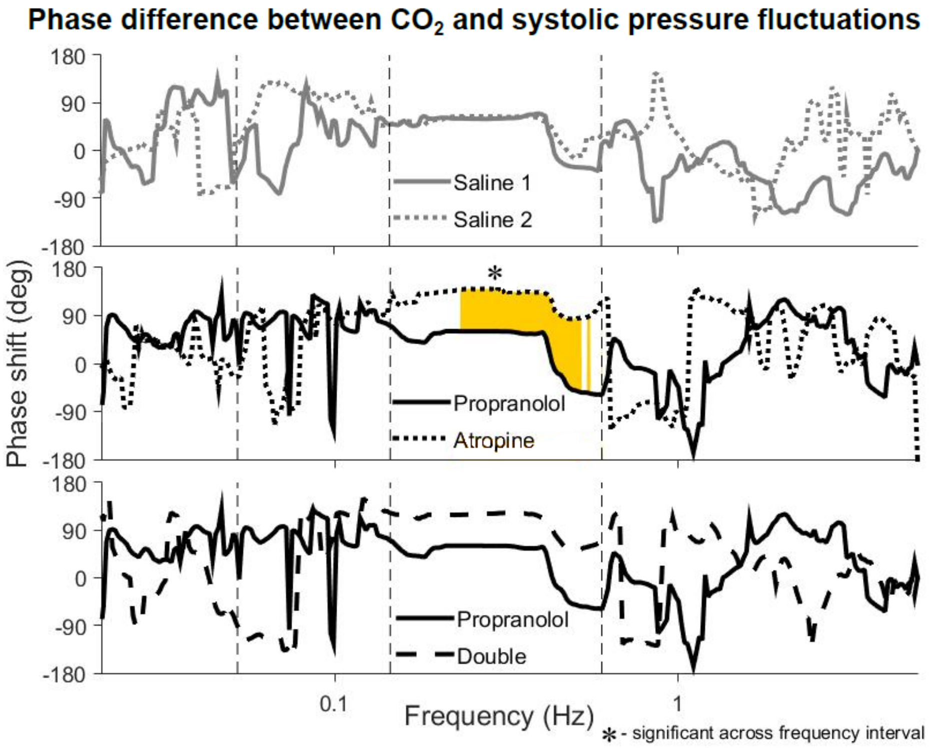

Figure S4. *Median phase shift in the wavelet phases of systolic blood pressure oscillations and respiration during fixed-frequency breathing.* The shifts are shown after atropine, propranolol and the double blockade as well as during the two saline controls. Rejection of the null hypothesis of the Friedman test, indicating significant phase shifts, are shown in yellow.

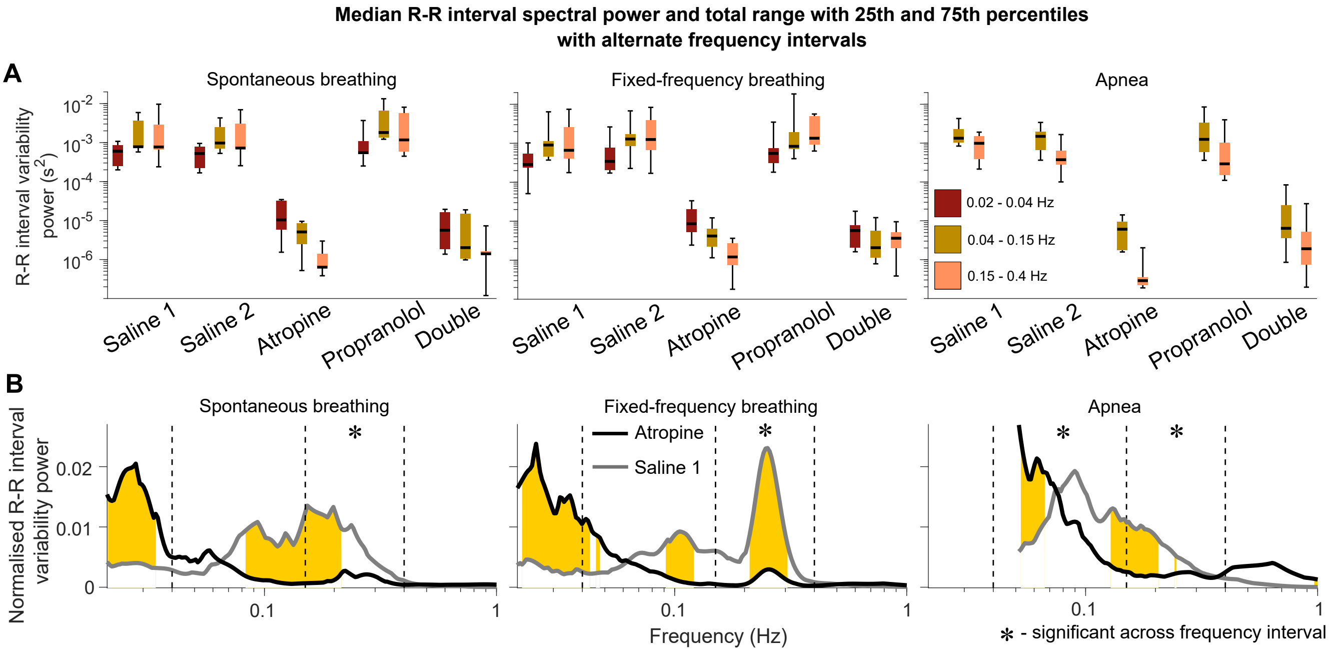

Figure S5. *Spectral analysis of the R-R interval signals with alternate frequency intervals.* The power in each frequency interval was found by time-averaging over the corresponding section of the wavelet transform. In **(A)** the median values are given by the black horizontal lines which intersect the colored boxes. The top and bottom of the boxes are located at the 75th and 25th percentiles respectively and the whiskers give the total range. In **(B)** the power distributions were normalised by dividing by the total power (sum of the curve) to compare the relative changes in the frequency distributions. The black and grey lines are the medians across all of the subjects. Data from the 0.02–0.04 Hz interval could not be determined for the apnea results due to the shortness of the measurements. Frequencies where the null hypothesis of the Friedman test was rejected for the saline 1 and atropine results are shown in yellow.

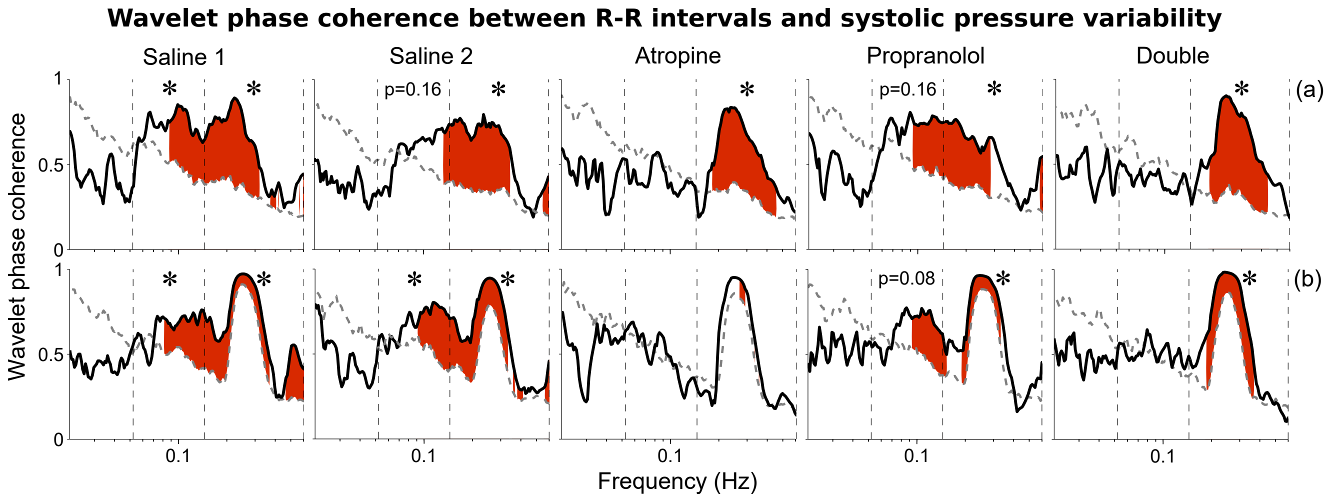

Figure S6. *Median wavelet phase coherence between R-R intervals and systolic blood pressure.* The median phase coherence is shown for: (a) spontaneous breathing, (b) fixed-frequency breathing. The solid black line is the median coherence across all subjects and the dashed grey line is the median surrogate level calculated from the distance of the coherence in each subject from the 95th percentile of the surrogates. The frequencies of significant coherence as identified by the Wilcoxon signed-rank test are shaded red. Significant coherence across the frequency interval is marked with *, while p-values are provided where there was some coherence in the interval but it was not significant.
